# Correction to: Nicotine inhibits activation of microglial proton currents via interactions with α7 acetylcholine receptors

**DOI:** 10.1007/s12576-018-0608-6

**Published:** 2018-04-20

**Authors:** Mami Noda, AI Kobayashi

**Affiliations:** Laboratory of Pathophysiology, Graduate School of Pharmaceutical Sciences, Kyushu University, 3-1-1 Maidashi, Higashi-ku, Fukuoka, 812-8582 Japan

## Correction to: J Physiol Sci (2017) 67:235–245 10.1007/s12576-016-0460-5

The article Nicotine inhibits activation of microglial proton currents via interactions with α7 acetylcholine receptors, written by Mami Noda and AI Kobayashi, was originally published Online First without open access. After publication in volume 67, issue 1, pages 235–245 the author decided to opt for Open Choice and to make the article an open access publication. Therefore, the copyright of the article has been changed to © The Author(s) 2018 and the article is forthwith distributed under the terms of the Creative Commons Attribution 4.0 International License (http://creativecommons.org/licenses/by/4.0/), which permits use, duplication, adaptation, distribution and reproduction in any medium or format, as long as you give appropriate credit to the original author(s) and the source, provide a link to the Creative Commons license and indicate if changes were made.

The original article was corrected.

